# Impact of Pretreatment of the Bulk Starting Material on the Efficiency of Liquid Phase Exfoliation of WS_2_

**DOI:** 10.3390/nano11051072

**Published:** 2021-04-22

**Authors:** Steffen Ott, Melanie Lakmann, Claudia Backes

**Affiliations:** Applied Physical Chemistry, Institute for Physical Chemistry, Faculty of Chemistry and Earth Sciences, Heidelberg University, Im Neuenheimer Feld 253, 69120 Heidelberg, Germany; steffen.ott@pci.uni-heidelberg.de (S.O.); Lakmann@stud.uni-heidelberg.de (M.L.)

**Keywords:** liquid phase exfoliation, 2D materials, optical properties, intercalation

## Abstract

Liquid phase exfoliation (LPE) is widely used to produce colloidal dispersions of nanomaterials, in particular two-dimensional nanosheets. The degree of exfoliation, i.e., the length to thickness aspect ratio was shown to be intrinsically limited by the ratio of in-plane to out-of-plane binding strength. In this work, we investigate whether simple pretreatment of the starting material can be used to change the in-plane to out-of-plane binding strength through mild intercalation to improve the sample quality in sonication-assisted LPE. Five different pretreatment conditions of WS_2_ were tested and the dispersions size-selected through centrifugation. From optical spectroscopy (extinction, Raman, photoluminescence), information on nanosheet dimension (average lateral size, layer number, monolayer size) and optical quality (relative photoluminescence quantum yield) was extracted. We find that the pretreatment has a minor impact on the length/thickness aspect ratio, but that photoluminescence quantum yield can be increased in particular using mild sonication conditions. We attribute this to the successful exfoliation of nanosheets with a lower degree of basal plane defectiveness. This work emphasizes the complexity of the exfoliation process and suggests that the role of defects has to be considered for a comprehensive picture.

## 1. Introduction

One versatile method to obtain two-dimensional nanosheets is liquid phase exfoliation (LPE), which was demonstrated for graphene in 2008 [1]. LPE can deliver dispersions of nanosheets with high yield and is applicable to a wide range of materials, including h-BN, [2,3] transition metal dichalcogenides (TMDs) in their 2H-polytype (MoS_2_ or WS_2_ [2,3,4]) or 1T-polytype (TaS_2_ [5] or ReS_2_ [6,7]), III-VI semiconductors (GaS [8], InSe [9]), oxides (MnO_2_ [10]), pnictogens (black phosphorus [11,12,13], Sb [14,15]), layered double hydroxides [16], naturally occurring minerals such as franckeite [17] or cylindrite [18], MXenes [19] or organic layered polymers [20] to name just a few. Liquid phase exfoliation is considered a two-step process involving mechanical delamination of the van der Waals crystals in liquid media through high energy processes such as sonication to overcome the interactions between the layers followed by subsequent colloidal stabilization in the liquid for example through the aid of suitable solvents or surfactants which prevent reaggregation. The resulting nanosheets can be further cast or printed into thin films and structures suitable for various applications ranging from nanocomposites, (opto)electronics and photonics to sensing and energy storage and conversion [21,22,23,24].

One downside of LPE is that exfoliated stock dispersions will always be polydisperse with a broad distribution in nanosheet size [25,26]. Centrifugation can be applied to narrow the size and thickness distributions [27]. For example, density gradient ultracentrifugation can be used to sort nanosheets dispersed in aqueous media by their buoyant density and hence layer number as demonstrated for graphene [28], h-BN [29], MoS_2_ [30] or ReS_2_ [6]. However, this technique is not frequently applied to produce high quality inks due to some restrictions such as low yield and the presence of a density gradient medium which is difficult to remove. In this regard, liquid-cascade centrifugation (LCC) [31] is an alternative, as it is applicable to a range of materials in both aqueous media and organic solvents. In this technique, large and thick nanosheets are efficiently separated from small and thin sheets.

In spite of the widespread use of LPE, the detailed mechanism of the process is topic of ongoing research [32,33,34,35] and not yet fully understood. For example, the role of defects in sonication-assisted LPE is still in debate. While liquid-exfoliated nanosheets are considered widely defect-free on the basal plane [34,36], a number of groups have found evidence for basal plane defects in the case of graphene [37,38,39]. In addition, it is typically observed that nanosheets thinned down to the monolayer (ML) limit are comparably small in their lateral dimensions (~100–200 nm for graphene, <50 nm for TMDs), despite of many attempts to optimize the exfoliation process [25,40,41,42,43,44]. In general, it is found that thin sheets tend to be small, while large sheets tend to be thicker [8,26,45]. Recently, a theoretical model has been developed, which suggests that this is because nanosheet exfoliation and scission go hand in hand in high energy LPE with an equipartition of energy [46]. The work demonstrated that the relationship between size and thickness of exfoliated nanosheets depends on the ratio of intra- and interlayer binding strength [46] as suggested earlier [47]. In a previous study we delivered further experimental support for this model and reported that the dimensions of MoS_2_ nanosheets obtained by LPE are not influenced by the particle size and quality of the starting material [48]. This means that the lateral size of ML nanosheets will be intrinsically limited by the material’s binding strength and that the resultant aspect ratio will depend on the crystal structure. Note that this picture of LPE also explains why it is also possible to exfoliate non van der Waals crystals with anisotropic binding situation, as demonstrated recently for metal diborides [49], silicon [50] or germanium [51].

The model also implies that it should be possible to shift the relationship between lateral size and thickness, i.e., increase the lateral size/thickness aspect ratio for a given van der Waals crystal if the binding strength is modified. This could for example be achieved by intercalation which reduces the interaction between the sheets. For example, the intercalation of 1-pyrenecarboxylic acid into graphite was suggested as efficient strategy within the context of surfactant-assisted LPE [52] with similar effects anticipated for TMDs [53]. Furthermore, chemical or electrochemical exfoliation, where intercalation is exploited, produce monolayers that are significantly larger than the LPE counterparts [54,55,56,57,58,59]. However, these chemical treatments can significantly alter the material properties and/or introduce lattice defects.

In this study, we investigate whether the material dependent length to thickness aspect ratio can be altered by simple pretreatment of the starting material prior to exfoliation with the aim to achieve intercalation of solvents or ions which might lower the interlayer binding strength. We subjected pretreated WS_2_ bulk material to sonication-assisted LPE and analyzed how potential intercalation changes the yield, nanosheet sizes and optical properties of the resulting nanosheets. We chose WS_2_ as a model system due to its rich optical properties that enable a straightforward assessment of nanosheet dimensions by established spectroscopic metrics.

## 2. Materials and Methods

### 2.1. WS_2_ Pretreatment

NaCl stir: 2.4 g WS_2_ were dispersed in an aq. NaCl-solution (1 M, 80 mL) and stirred at room temperature (RT) for 8 days. The dispersion was filtered using a vacuum filtration setup with a nitrocellulose membrane (MF^TM^—membrane filters from Merck Millipore LTd., Tullagreen, Carrigtwohill Co. Cork, Ireland, 0.025 µm pore size, 47 mm diameter) and thoroughly washed with water. The WS_2_ powder was carefully scraped off and dried under vacuum. The dried powder was weighed to determine the amount of starting material for the calculation of the yield.

NMP stir: 2.4 g WS_2_ were dispersed in 80 mL of NMP and stirred at room temperature for 7 days. The dispersion was filtered and dried under vacuum, as described above in detail.

*n*-BuLi stir: 2.4 g WS_2_ was dispersed in 20 mL of dry *n*-hexane and *n*-Buli (0.6 mL, 2 M) was added. The solution was stirred overnight at 60 °C and quenched with 100 mL of water. WS_2_ accumulated in the aqueous phase, which was washed with *n*-hexane. After filtration, the WS_2_ powder was washed with water and dried under vacuum, as described above in detail.

H_2_O bath: 2.4 g WS_2_ was dispersed in 80 mL of water in a round bottom flask and sonicated for 7 h in a sonication bath (Bransonic^®^ CPXH 2800-E, Branson Ultrasonics Corporation, Danbury, CT, USA). The filling height in the sonication bath was adjusted in such a way that hot spots formed and the round bottom flask was placed in the middle of the hot spot. Ice was successively added to the sonication bath to avoid heating of the water, which required continuous re-adjustment of the filling height. After sonication the dispersion was filtered and dried under vacuum, as described above in detail.

LiCl bath: 2.4 g WS_2_ was dispersed in a solution of 0.41 g LiCl in 20 mL *n*-hexane and sonicated for 4 h in a sonication bath. The dispersion was filtered, thoroughly washed with water and dried under vacuum, as described above in detail.

### 2.2. Exfoliation and Size Selection

Liquid phase exfoliation was executed by tip sonication using a Sonics VCX 500 (Sonics & Materials, Inc., Newtown, CT, USA, 500 W) tip sonicator equipped with a horn tip (1.27 cm diameter) for 500 W ultrasonic processor from Sigma Aldrich (St. Louis, MO, USA). The WS_2_ powder obtained after the pretreatment (≈1.8–2.4 g) was dispersed in 80 mL of an aqueous sodium cholate (SC) solution (2 g/L) and sonicated for 5 h at 5 °C external temperature (pulse: 6 s on, 4 s off, amplitude 30% or 60%, respectively) in a metal beaker. The exfoliated stock dispersion was size-selected by liquid cascade centrifugation in a Hettich MIKRO 220R centrifuge (Andreas Hettich GmBH & Co. KG, Tuttlingen, Germany) equipped with a fixed angle rotor 1016 or 1195-A at 10 °C and in centrifugation steps of 2 h. After a first centrifugation step at a relative centrifugal acceleration of 0.1 k *g* (100 *g*, where *g* is the earth’s gravitational field) the sediment was discarded to remove any unexfoliated material. The supernatant was successively centrifuged again at 0.4, 0.8, 2, 5, 10 and 30 k *g* and the sediments collected and redispersed in a defined volume of aq. SC-solution (0.1 g/L). Centrifugations from 0.1 to 2 k *g* were carried out in 50 mL tubes (VWR high performance, conical bottom centrifuge tubes) with a filling height of 20 mL in the rotor 1016. From 5 k *g* on, centrifugations were carried out in aliquots of 1.5 mL in Eppendorf tubes in the 1195-A rotor. The sediment collected after 0.4 k *g* was labelled as 0.1–0.4 k *g*, the sediment collected after 0.8 k *g* was labelled as 0.4–0.8 k *g*, and so on. The supernatant of the centrifugation step at 30 k *g* was discarded.

### 2.3. Characterization

UV Vis extinction spectroscopy was carried out by a Varian Cary 6000i UV/Vis/NIR spectrometer (Agilent Technologies, Inc., Santa Clara, CA, USA). The collected WS_2_ samples were diluted in an aqueous SC solution (0.1 g/L), measured in a Quartz SUPRASIL^®^ cuvette (Hellma GmbH & Co. KG, Müllheim, Germany) with a 4 mm beam path and the baseline was subtracted. To determine the position of the A exciton, the spectra were normalized to the extinction at 294 nm and the second derivative, d^2^Ext/dE^2^, was calculated. The derived spectra were smoothed by adjacent averaging with averaging 30 points and the exciton position was read out. This procedure partially compensates artificial shifts caused by the scattering background.

Raman spectra were collected by a Renishaw InVIA confocal Raman microscope (Renishaw plc, Wotton-under-Edge, Gloucestershire, UK) with a 532 nm excitation laser. The microscope was equipped with a 50× long working distance objective lens in streamline mode to collect the Raman emission and a 2400 L/mm grating to disperse the emission. For the measurements, concentrated WS_2_ dispersions were dropped on aluminum foil. The laser was focused to the surface of the droplet, since focusing inside the droplet lowers the PL intensity due to innerfilter and reabsorption effects. 5–10 spots of each droplet were measured under ambient conditions with 1% of the laser power. The spectra were collected in the edge region of the droplet, where the height and focusing plane is less effected by evaporation of water that occurs during the measurement. After acquisition the spectra were averaged, baseline corrected and normalized to the 2LA(M) Raman mode.

## 3. Results

**Figure 1:**
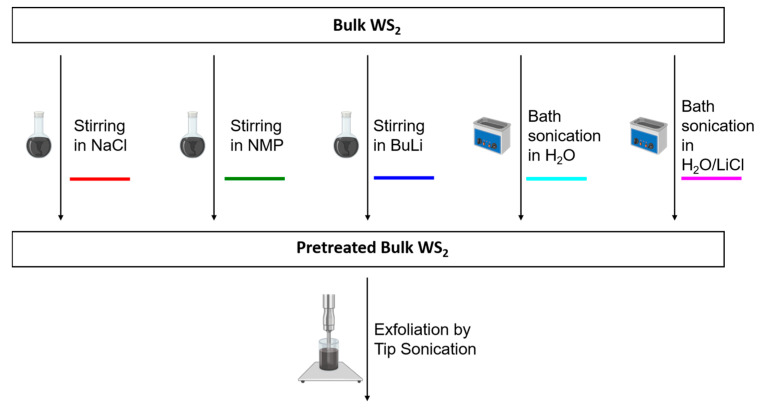
Pretreatment scheme of WS_2_. Bulk WS_2_ was subjected to 5 different pretreatments prior to exfoliation, including stirring in NMP, stirring in aqueous NaCl solution, stirring in hexane/BuLi, bath sonication in water and bath sonication in an aqueous LiCl solution.

Bulk WS_2_ was treated with five different methods (Figure 1) including simple approaches such as stirring in aqueous NaCl solution (NaCl stir), stirring in NMP (NMP stir), and bath sonication in H_2_O (H_2_O bath) as well as methods that are reported to result in intercalation such as stirring in *n*-BuLi solution (BuLi stir) and bath sonication in aq. LiCl solution (LiCl bath) [60]. See experimental section for details. The pretreated materials were filtered, excessively washed to remove the pretreatment agents and dried under vacuum. The dried WS_2_ powders were dispersed in 80 mL of an aqueous sodium cholate solution (SC, 2 g/L) and exfoliated by tip sonication. Our standard exfoliation protocol includes tip sonication at an amplitude of 60%. However, in this study we first performed the sonication with a comparably low amplitude of 30% to avoid that potentially small impacts of the pretreatments become negligible under harsh sonication conditions that deliver more energy. As will be shown below, we observed some differences between the dispersions exfoliated from the pretreated starting materials and the reference samples. Therefore, the pretreatment of stirring in NaCl was repeated and followed by an exfoliation with a sonication amplitude of 60% (NaCl stir 60%) to see if the impact of the pretreatment still remains when using our high energy standard exfoliation protocol.

For the ease of characterization, we subjected the stock dispersions to liquid cascade centrifugation (LCC) for size selection in analogy to previous reported procedures [31]. In LCC, a dispersion is successively centrifuged with increasing centrifugal accelerations (expressed as relative centrifugal force, RCF, expressed in multiples of the earth’s gravitational field *g*). By collecting the sediments after each step, dispersions containing different nanosheet sizes are obtained. The sediment of the first centrifugation step at 0.1 k *g* (100 *g*) was discarded since it contains predominantly unexfoliated material, while the other sediments were collected in a reduced volume of fresh solution (0.1 g/L SC). For each dispersion, six fractions were isolated at 0.4, 0.8, 2, 5, 10 and 30 k *g*, where the first sediment, collected after 0.4 k *g*, is labelled as 0.1–0.4 k *g*, the second sediment collected after 0.8 k *g* is labelled as 0.4–0.8 k *g* and so on.

**Figure 2:**
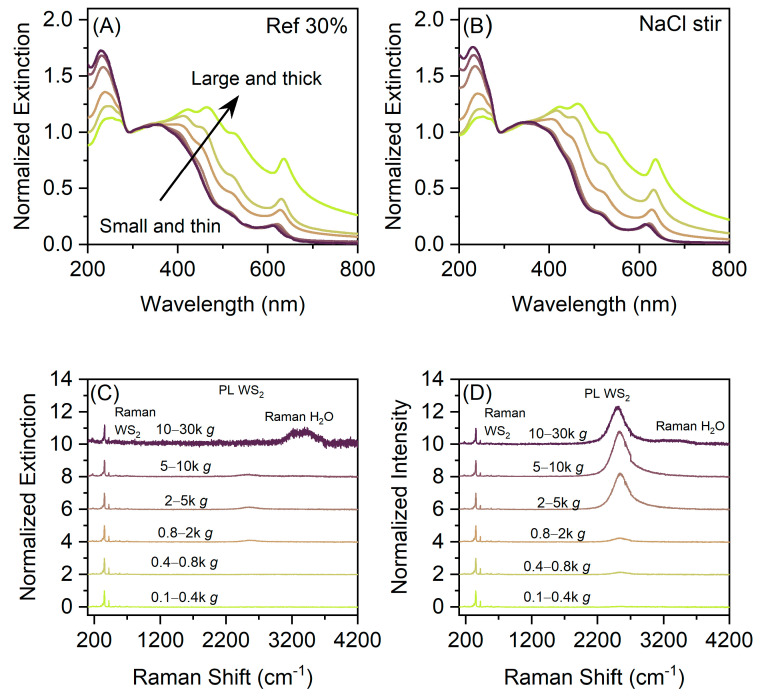
Spectroscopic characterization of the size-selected dispersion. (**A**,**B**) Normalized extinction spectra of the reference exfoliated with an amplitude of 30% (**A**) and the sample set corresponding to the WS_2_ exfoliated with 30% amplitude after pretreating the powder by stirring in NaCl (**B**). (**C**,**D**) Normalized Raman spectra (532 nm excitation) of the reference (**C**) and NaCl-pretreated sample (**D**). Spectra are offset for clarity.

The sediments were subjected to UV/Vis extinction spectroscopy (Figure 2A,B), as well as Raman spectroscopy (Figure 2C,D). For all data, see Appendix A. All extinction spectra feature the characteristic excitonic transitions of 2H-WS_2_ [61]. Within one sample set, the extinction spectra show characteristic size-dependent spectral changes (arrow in Figure 2A), which mostly originate from both changes in the absorbance and the scattering component of the extinction [31,45]. In a first approximation the optical extinction spectra of small nanosheets can be considered as equal to the absorbance spectra and changes mostly arise from edge effects, where the electronic structure is distinct to the basal plane. The extinction spectra of larger nanosheets contain significant contributions of a scattering background which leads to an increased extinction in the non-resonant regime (at excitation energies below the onset of absorbance, >650 nm). The resonant regime of these extinction spectra is dominated by the absorbance, which allows to calculate the nanosheet concentration of a dispersion directly from the extinction, Ext, by the Beer–Lambert law, Ext_235_ = ε_235_ c d, where ε_235_ = 47.7 lg^−1^cm^−1^ [31] is the extinction coefficient at 235 nm, c the concentration of the dispersion and d the path length of the cuvette.

In addition, as shown in previous work [4,31,45], the relative contributions of absorbance and scattering are not the only origin of the observed spectral changes. Edges are electronically different compared to basal planes and have a different absorbance coefficient at each wavelength. Therefore, peak intensity ratios can be used to determine the average lateral size of the nanosheets. The mean nanosheet length, <L>, can be calculated from the ratio of the extinction at 235 nm, Ext_235_, to the extinction at 290 nm, Ext_290_, according to Equation (1) [31]. Furthermore, confinement and dielectric screening effects have an impact on the exciton energies, i.e., peak positions [4,62]. The mean layer number, <N>, can be calculated from the position of the A exciton by equation 2 [31]. In analogy to previous work [4,31] we determined the position of the A exciton from the second derivative of the spectra, d^2^Ext/dE^2^, since this will partially compensate for artificial shifts caused by the scattering background.

(1) $$\langle L\rangle =\frac{2.30-{\mathrm{Ext}}_{235}/{\mathrm{Ext}}_{290}}{0.02{\mathrm{Ext}}_{235}/{\mathrm{Ext}}_{290}-0.0185}$$

(2) $$\langle N\rangle =6.35\cdot 10^{-32}\cdot \exp\left(\lambda_{A}\left(\mathrm{nm}\right)/8.51\right)$$

**Figure A1:**
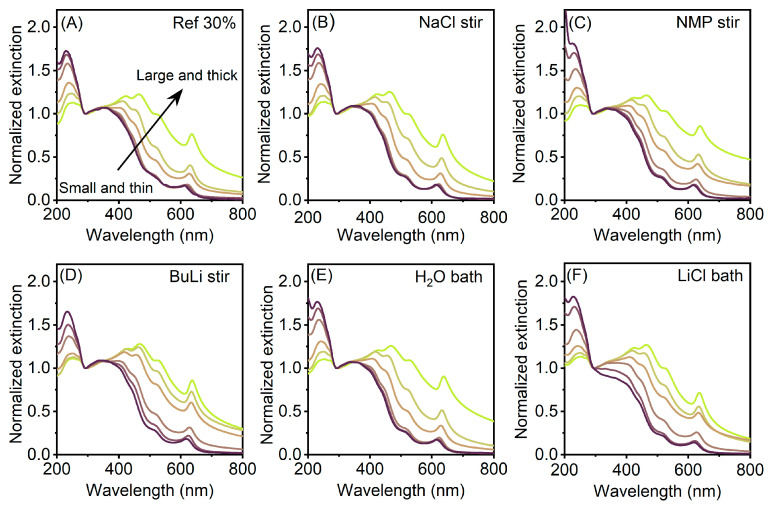
Optical extinction spectra of liquid-phase exfoliated and size selected WS_2_ nanosheet dispersions. The bulk WS_2_ powder was subjected to the following pretreatments, prior to exfoliation: (**A**) Reference, (**B**) stirring in H_2_O/NaCl, (**C**) stirring in NMP, (**D**) stirring in hexane/*n*-BuLi, (**E**) bath sonication in H_2_O, (**F**) bath sonication in an H_2_O/LiCl. The exfoliation was executed by tip sonication with an 30% amplitude. Size dependent spectral changes are indicated by the arrow in (**A**).

**Figure A2:**
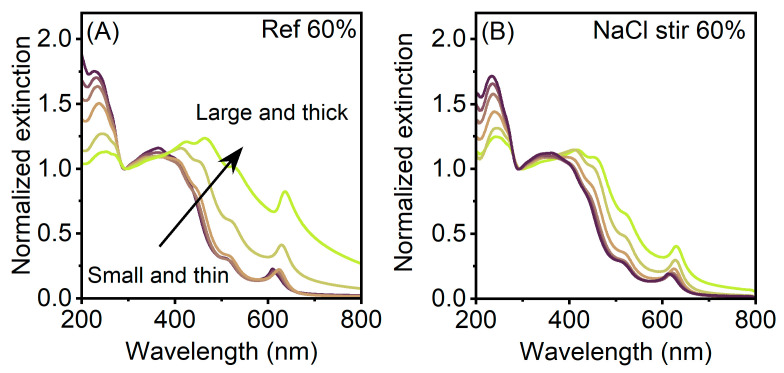
Optical extinction spectra of liquid-phase exfoliated and size selected WS_2_ nanosheet dispersions. The exfoliation was executed by tip sonication with an 60% amplitude. (**A**) Reference, (**B**) Pretreatment of the bulk starting material by stirring in H_2_O/NaCl. Size dependent spectral changes are indicated by the arrow in (**A**).

While the extinction spectra of the reference and the pretreated samples (Figure 2A,B, Figure A1 and Figure A2) only show minor variations with pretreatment at the cursory glance, this analysis is capable of revealing even subtle effects as will be discussed below. We note that samples will still be polydisperse after cascade centrifugation [31]. However, the standard errors of the averages, which is the relevant quantity in this case, can be assessed with reasonable accuracy. The overall error of the determination of the average length and thickness from optical extinction spectra was recently evaluated in context of reference [25] and found to be 6% for <L> and 13% for <N>.

**Figure A3:**
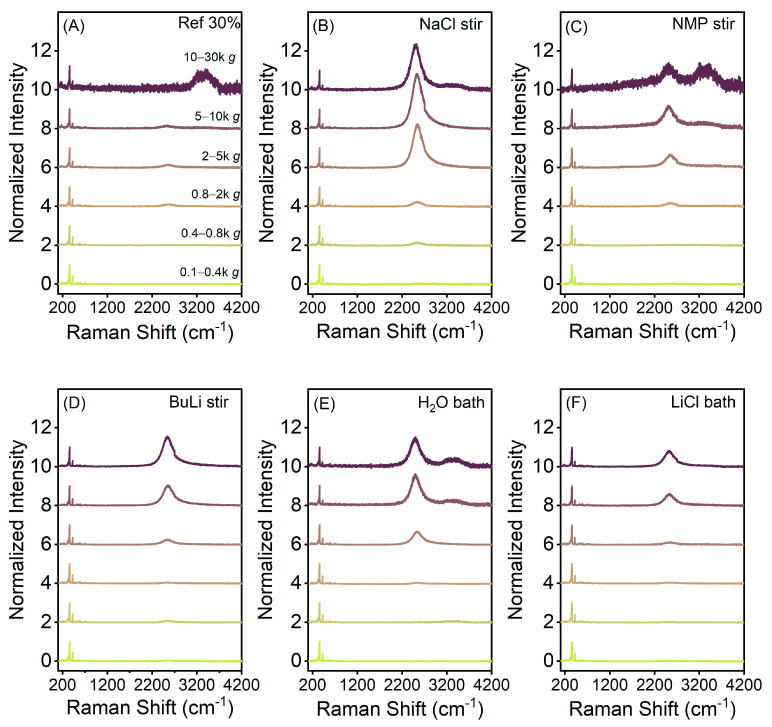
Raman spectra of WS_2_ nanosheet dispersions, exfoliated by tip sonication with 30% amplitude. The bulk WS_2_ powder was subjected to the different pretreatments, prior to exfoliation. (**A**) Reference, (**B**) stirring in H_2_O/NaCl, (**C**) stirring in NMP, (**D**) stirring in hexane/*n*-BuLi, (**E**) bath sonication in H_2_O, (**F**) bath sonication in an H_2_O/LiCl. The spectra display the characteristic Raman signals of WS_2_. The PL of WS_2_ is displayed around 2500 cm^−1^ and negligible in dispersions containing large nanosheets but increases with the ML content of the dispersion. Signals around 3400 cm^−1^ originate from water. The spectra of the different nanosheet sizes are vertically offset for clarity.

**Figure A4:**
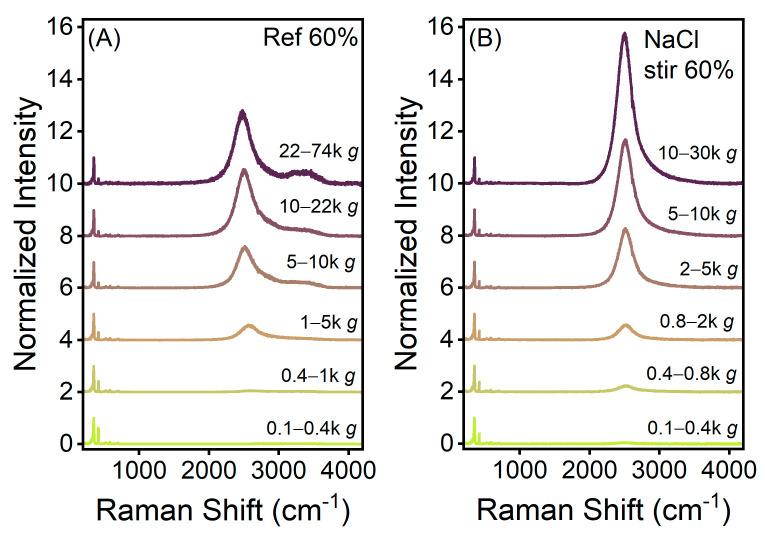
Raman spectra of liquid-phase exfoliated WS_2_ nanosheet dispersions. Exfoliation was executed by tip sonication with a 60% amplitude. The spectra display the characteristic Raman signals of WS_2_. The PL of WS_2_ is displayed around 2500 cm^−1^ and negligible in dispersions containing large nanosheets but increases with the ML content of the dispersion. Signals around 3400 cm^−1^ originate from water. (**A**) Reference, (**B**) Stirring in H_2_O/NaCl. The spectra of the different nanosheet sizes are vertically offset for clarity.

To evaluate the output of LPE, not only the dimensions of the nanosheets are a matter of interest, but also the optical properties, in particular the photoluminescence (PL) which can be regarded as a measure for sample quality. For example, as shown previously [31] only mono-layered WS_2_ with reasonable lateral size (>25 nm) will exhibit the characteristic excitonic emission of the direct bandgap monolayers. In addition, we expect that lattice defects will broaden or quench the photoluminescence [63]. The fluorescence spectra were measured on concentrated sediments after LCC in a Raman spectrometer (excitation wavelength 532 nm). This procedure has the advantage that the WS_2_ Raman and PL are measured simultaneously. Previous work suggested that the PL/Raman ratio is predominantly a measure for the monolayer content [31] with negligible influence from the surrounding stabilizer [25]. However, it should be noted that the role of basal plane defects on the PL of LPE TMDs has not been studied in detail and it is likely that the PL quantum yield will also depend on the defectiveness of the material. The Raman/PL spectra of the reference as well as the sample set exfoliated after pretreatment by stirring in NaCl are shown in Figure 2C,D. All data see appendix, Figure A3 and Figure A4. In all batches an increase of the PL is observed for samples collected at higher centrifugal acceleration, i.e., with decreasing average nanosheet thickness and therefore higher monolayer content. However, significant differences in the PL intensities between the batches are observed. For example, the reference sample set exfoliated with 30% amplitude of the sonicator shows only very weak PL suggesting that either no monolayers are present or that these are either laterally small or highly defective. This will be analyzed in more detail and discussed in context with the size information from the extinction spectra.

## 4. Discussion

**Figure 3:**
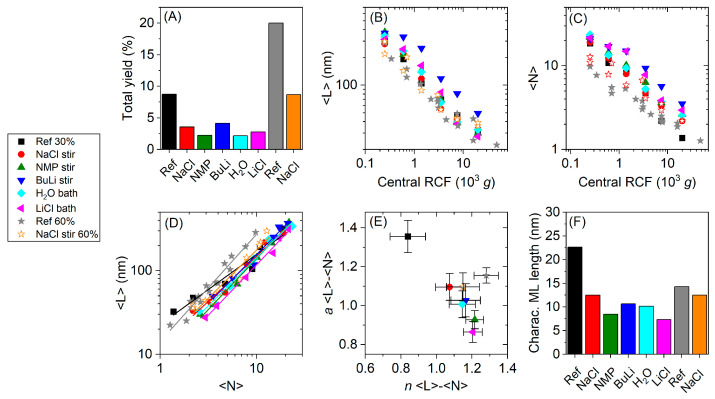
Yield and size information of LPE WS_2_ nanosheets, derived from the optical extinction spectra in Figure A1 and Figure A2. The bulk WS_2_ powders were subjected to different pretreatments prior to exfoliation and the exfoliated stock dispersions were size selected by liquid-cascade centrifugation. (**A**) Total yield of the different batches. The total yield is the sum of the individual yields of each fraction obtained during the size selection process, excluding the sediment of the first centrifugation step, which was discarded. The yield of the pretreated materials is lower than the references. (**B**) Mean nanosheet length, <L>, as a function of central RCF. (**C**) Mean nanosheet layer number, <N> as a function of central RCF. (**D**) <L> as a function of <N>, fitted by power laws. (**E**) Prefactor and exponents extracted from the power law fits in (**D**). Pretreated materials group together while Ref 30% exhibits a higher prefactor and lower exponent. (**F**) Characteristic ML length of the different batches. Highest ML length was found for Ref 30%.

The information on yield, nanosheet lateral size and layer number extracted from the extinction spectra is summarized in Figure 3. The total yield, i.e., the sum of the individual yields of each fraction collected after the size selection is illustrated in Figure 3A. The total yield of Ref 30% is with 9% the highest yield within the 30% amplitude series. The yields of pretreated materials show some variations in the order H_2_O bath < NMP stir < LiCl bath < NaCl stir < BuLi stir, but all of them are less than half of the yield of the reference sample. The yield of Ref 60% is approximately doubled compared to Ref 30%. Due to the higher energy input during the sonication with 60% amplitude, a larger portion of the bulk material is dispersed/exfoliated at identical processing times in agreement with previous findings [42]. Again, the yield of the pretreated NaCl stir 60% is only half of the Ref 60% yield, indicating that the pretreatment generally decreases the yield. While this points to an unfavorable exfoliation after pretreatment, the decrease in yield cannot be easily rationalized. A possible reason might be incomplete removal of the pretreatment agents in the washing step. However, it is extremely unlikely that this accounts for ~1/2 of the mass of the starting material due to the significantly lower molecular weight of the pretreatment agents compared to WS_2_. For example, even in the case of exfoliated graphene nanosheets in aqueous surfactant, the surfactant was found to make up <20% of the mass [64]. Another explanation approach could be the different amounts of starting materials, which result from losses during the pretreatments (see Section 2.1 for details) and changes the WS_2_ concentration and WS_2_/SC ratio. Considering that differences in the batch sizes are small, this is likely a minor factor. The decrease in yield currently remains a mystery.

While the yield is an important parameter in LPE, the aim of the study was to investigate whether the pretreatment has an impact on the length/thickness aspect ratio and/or optical quality of the WS_2_ nanosheets, such as photoluminescence quantum yield. In Figure 3B,C, the average lateral size <L> (Figure 3B) and nanosheet layer number, <N> (Figure 3C) are plotted against the midpoint of the centrifugation boundaries, central RCF. It was empirically found that these data typically follow a power law, resulting in a linear curve progression on a log-log scale [31]. The data points from the exfoliation runs at 60% amplitude are located below the 30% amplitude data points, i.e., at smaller <L> and <N> which is explained by the higher energy input during the exfoliation, resulting in a more proceeded exfoliation and higher populations of smaller and thinner nanosheets.

Figure 3D shows <L> as a function of <N>. Again, a powerlaw scaling is observed in agreement with previous reports [46,48]. Power law fits were applied in the form of Equation (3).

(3) $$\langle L\rangle =10^{a}\cdot {\langle N\rangle }^{n}$$

From these fits, we extracted the prefactor, 10*^a^*, and the exponent, *n*, which show significant variations across the batches, reflected in different slopes on the log-log scale. The results of the fits are displayed in Figure 3E, where the prefactors of the fits are plotted as a function of the exponent. In this plot the pretreated materials of the 30% amplitude series group together, but the untreated 30% amplitude (Ref 30%) exhibits a significant larger prefactor and smaller exponent. Ref 60% and NaCl stir 60% group together with the pretreated materials of the 30% series, even though with slightly higher prefactors. While the physical meaning of the exponent cannot be easily rationalized and reflects subtle difference in the mechanism of the exfoliation, the prefactor of the fits has a straightforward physical meaning, as it represents the extrapolated mean nanosheet length of the WS_2_ monolayers in dispersion (at <N> = 1), which we denote the characteristic ML length. The characteristic ML length is compared in Figure 3F and shows some unintuitive trends. First, with 23 nm, Ref 30% has a significantly higher characteristic ML length than the pretreated materials. Within the pretreated batches of the 30% amplitude series, the ML length rises in the order LiCl bath < NMP stir < H_2_O bath < BuLi stir < NaCl stir. Second, the characteristic ML length of Ref 30% exceeds the ML length of Ref 60%. According to the current understanding of the LPE process, the length to thickness ratio is a material dependent parameter and a result of in-plane and out-of-plane binding energies [46], as mentioned in the introduction of this paper. Based on this model, significant variations in the characteristic ML length are only expected if the out-of-plane binding energies are altered, what we take as an indication for successful intercalation of the pretreatment agents. However, intuitively one would expect that pretreatment and intercalation lead to increased lateral dimensions of the nanosheets. Furthermore, one would expect that Ref 30% and Ref 60% exhibit similar characteristic ML lengths, since no pretreatment was carried out and the characteristic monolayer length should be a result of the ratio of interlayer and intralayer binding strength in pristine WS_2_. This is in stark contrast to the experimental observations which suggest that additional factors have an impact on the length/thickness aspect ratio.

The expectations outlined above are based on a relatively minimal model [46] which does not consider the role of defects, which are likely always present in practice. We assume that defects have an impact on the ratio of the interlayer to intralayer binding strength. As such, one could rationalize the changes in the characteristic monolayer length through variations in the degree of basal plane defectiveness. As will be shown in context with the PL measurements below, the nanosheets in the 30% reference batch with a larger characteristic monolayer length are more defective than the nanosheets in the pretreated samples, as they exhibit a lower relative quantum yield. This implies that defective sheets have a lower out of plane binding strength and that less energy is required to achieve their exfoliation. At the same time, assuming equipartition of energy, scission events are also reduced which results in an increase in the characteristic ML length.

With this in mind, we can now turn to an interpretation of the pretreatment. If, despite of the low amplitude, pretreatment and intercalation additionally lowers the interlayer binding energy, this should allow for the exfoliation of defect-free areas. However, it is possible that the exfoliated nanosheets with low defect content will be laterally smaller than defect-rich nanosheets. A similar rational can be used to explain the difference between the ML length of Ref 30% and Ref 60%. The high energy input during the exfoliation of Ref 60% allows the exfoliation of defect-free areas, which will result in laterally smaller sheets. Interestingly the ML length of Ref 60% is larger than the ML length of the pretreated materials of the 30% amplitude series. This might suggest effective scission along basal plane defects, which results in an increase of the ML length. Within the 60% amplitude series, it was observed that NaCl stir 60% exhibits a smaller characteristic ML length than Ref 60%, even though the trend is less pronounced as in the 30% series.

**Figure 4:**
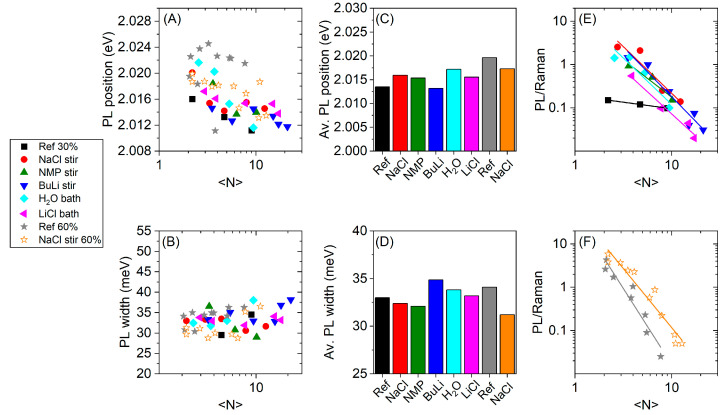
Analysis of the fluorescence data of liquid-phase exfoliated and size-selected WS_2_ nanosheets in aqueous dispersion, obtained from the Raman spectra (Figure A3 and Figure A4) and the corresponding fits shown in Figure A5, Figure A6, Figure A7, Figure A8, Figure A9, Figure A10, Figure A11 and Figure A12. (**A**) PL position as a function of <N>, (**B**) PL width as a function of <N>, (**C**) Average PL position of the different batches, (**D**) Average PL width of the different batches. In (**C**,**D**) the PL position and width were averaged over all collected samples of one pretreatment method. (**E**) PL/Raman ratios of the 30% amplitude series as a function of <N>. (**F**) PL/Raman ratios of the 60% amplitude series as a function of <N>. The PL/Raman ratios of the pretreated batches are higher than the references.

**Figure A5:**
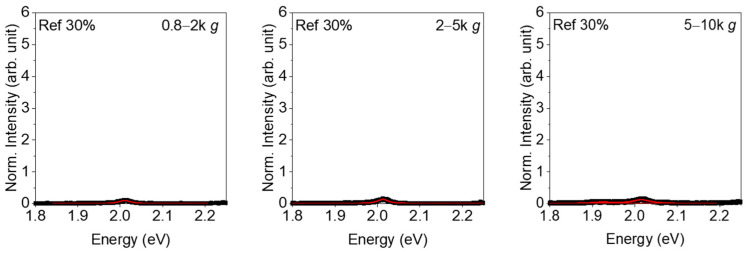
Photoluminescence spectra (λ_exc_ = 532 nm) of the reference WS_2_ nanosheet dispersions, exfoliated by tip sonication with a 30% amplitude. The PL was fitted by Lorentzians for quantitative analysis. Spectra of fractions which were collected during liquid cascade centrifugation but did not show any PL are excluded.

**Figure A6:**
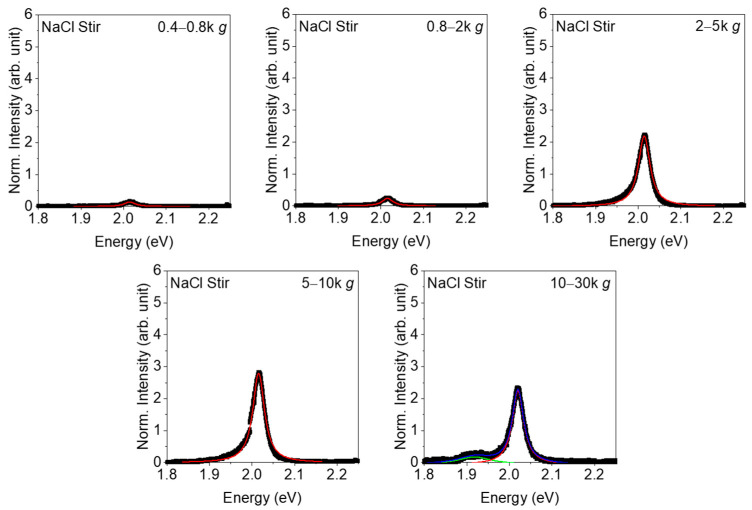
Photoluminescence spectra (λ_exc_ = 532 nm) the WS_2_ nanosheet dispersions, exfoliated by tip sonication with a 30% amplitude. The bulk starting material was stirred in H_2_O/NaCl prior to exfoliation. The PL was fitted by Lorentzians for quantitative analysis. Spectra of fractions which were collected during liquid cascade centrifugation but did not show any PL are excluded.

**Figure A7:**
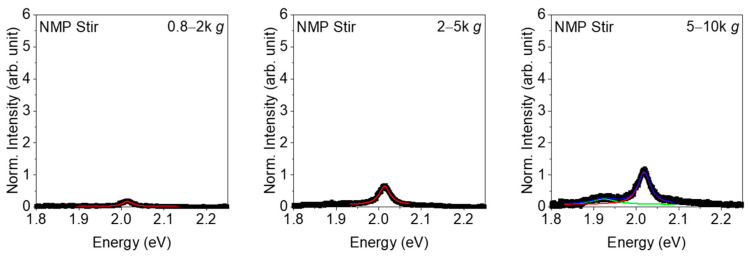
Photoluminescence spectra (λ_exc_ = 532 nm) the WS_2_ nanosheet dispersions, exfoliated by tip sonication with a 30% amplitude. The bulk starting material was stirred in NMP prior to exfoliation. The PL was fitted by Lorentzians for quantitative analysis. Spectra of fractions which were collected during liquid cascade centrifugation but did not show any PL are excluded.

**Figure A8:**
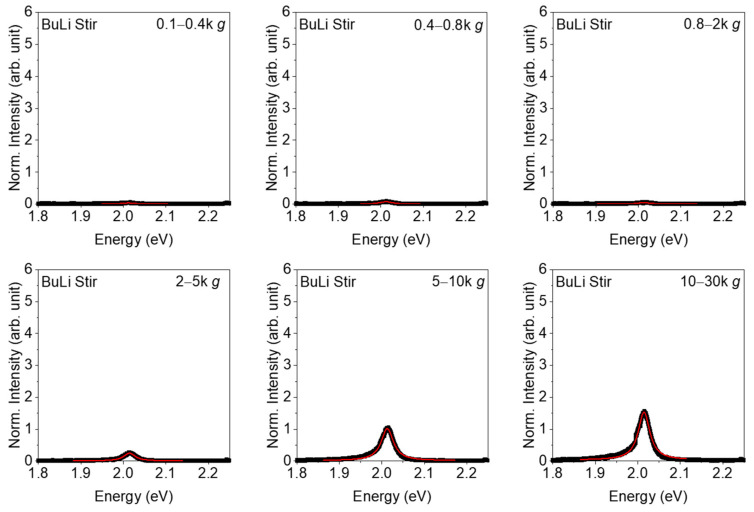
Photoluminescence spectra (λ_exc_ = 532 nm) the WS_2_ nanosheet dispersions, exfoliated by tip sonication with a 30% amplitude. The bulk starting material was stirred in hexane/*n*-BuLi prior to exfoliation. The PL was fitted by Lorentzians for quantitative analysis.

**Figure A9:**
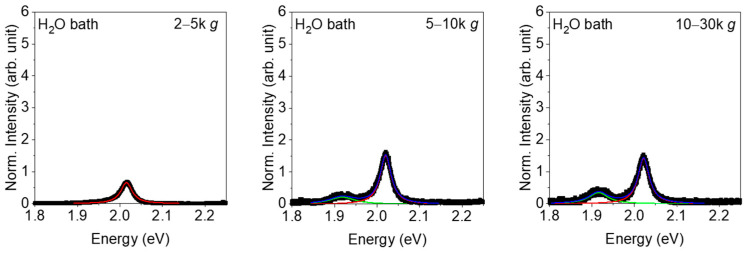
Photoluminescence spectra (λ_exc_ = 532 nm) the WS_2_ nanosheet dispersions, exfoliated by tip sonication with a 30% amplitude. The bulk starting material was bath sonicated in H_2_O prior to exfoliation. The PL was fitted by Lorentzians for quantitative analysis. Spectra of fractions which were collected during liquid cascade centrifugation but did not show any PL are excluded.

**Figure A10:**
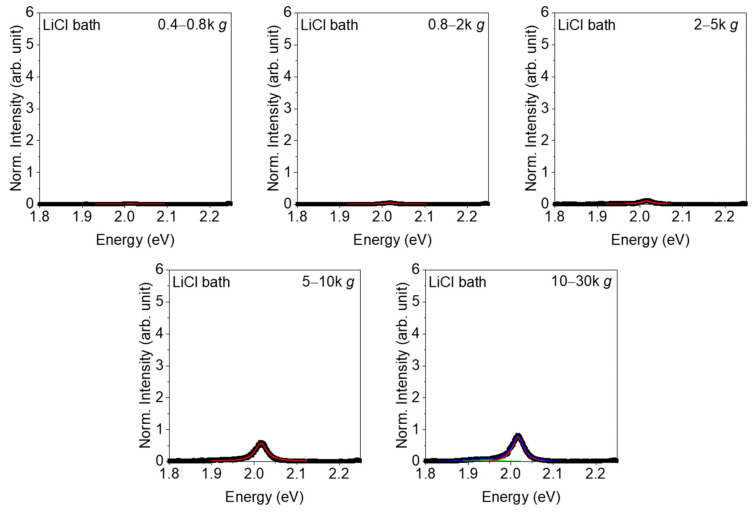
Photoluminescence spectra (λ_exc_ = 532 nm) the WS_2_ nanosheet dispersions, exfoliated by tip sonication with a 30% amplitude. The bulk starting material was bath sonicated in H_2_O/LiCl prior to exfoliation. The PL was fitted by Lorentzians for quantitative analysis. Spectra of fractions which were collected during liquid cascade centrifugation but did not show any PL are excluded.

**Figure A11:**
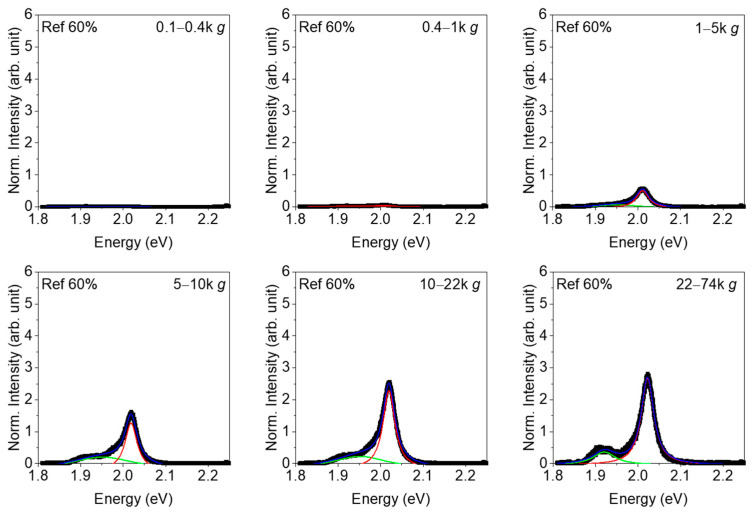
Photoluminescence spectra (λ_exc_ = 532 nm) of the reference WS_2_ nanosheet dispersions, exfoliated by tip sonication with a 60% amplitude. The PL was fitted by Lorentzians for quantitative analysis.

**Figure A12:**
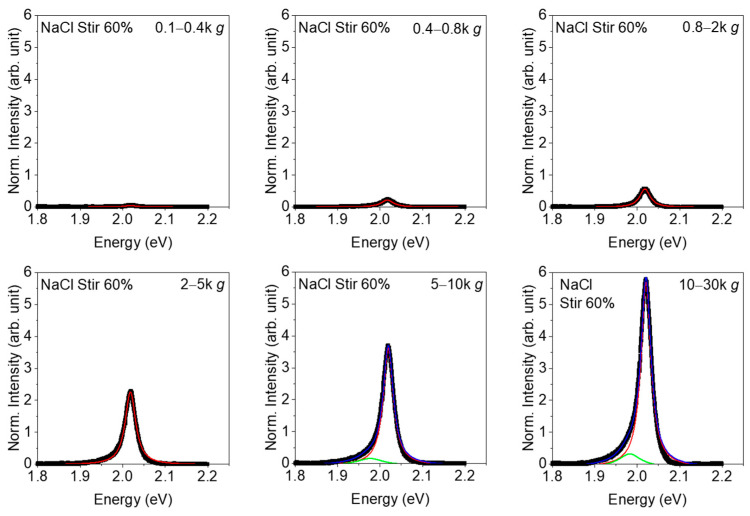
Photoluminescence spectra (λ_ex_c = 532 nm) the WS_2_ nanosheet dispersions, exfoliated by tip sonication with a 60% amplitude. The bulk starting material was stirred in H_2_O/NaCl prior to exfoliation. The PL was fitted by Lorentzians for quantitative analysis.

A simple way to test this hypothesis is through an assessment of the relative photoluminescence quantum yield which can be achieved by analyzing the Raman/PL spectra. Defective nanosheets are expected to exhibit a different PL response [63] as will be discussed in more detail below. Therefore, we analyze the Raman/PL spectra shown in Figure 2C,D, Figure A3 and Figure A4 in more detail. In most cases, the PL can be described well through a single Lorentzian (Figure A5, Figure A6, Figure A7, Figure A8, Figure A9, Figure A10, Figure A11 and Figure A12) and a component for the water Raman signal. The PL position and width were extracted from the fits. Figure 4A,B show the PL position and the PL width as a function of <N> for all sets of samples. The PL positions range from 2.012 eV (616 nm) and 2.024 eV (612.5 nm) consistent with excitonic emission of WS_2_ monolayers [65,66]. Overall, a redshift of the emission in fractions isolated at lower RCF containing on average larger/thicker sheets is observed (Figure 4A). The reason for this trend is currently unclear, but it could be a manifestation of varying defectiveness on the basal plane or possible energy transfer with few-layer sheets (e.g., in incompletely exfoliated sheet stacks with protruding monolayers). Overall, variations across the different batches are minor, as illustrated by the histograms in Figure 4C. No clear trends are discernible, except that the average WS_2_ PL of the sheets in the sample Ref 30% is redshifted compared to the pretreated samples and the exfoliation with 60% amplitude. In the PL width, no trend with the nanosheet layer number is observed (Figure 4B) and the majority of the samples show a room temperature PL linewidth between 30–35 meV in agreement with previous reports [4,31]. The average linewidth is similar across the different batches as illustrated by the histograms in Figure 4D. The batch NaCl stir 60% shows a slightly sharper PL which hints towards a better optical quality of the monolayers. However, the overall variation in linewidth and position is minor.

It is possible that defects on the nanosheet basal plane act as nonradiative decay channels reducing the photoluminescence quantum yield (PLQY) of the monolayers, but not necessarily changing PL position and width. It is thus important to access this information. While it is extremely challenging to determine absolute quantum yields of such relatively weakly emitting systems with sufficient precision, comparisons across different batches are possible by extracting the PL/Raman intensity or area ratio. This ratio will be affected by the monolayer content on the one hand [31] and the PLQY of the monolayer on the other hand. Since the monolayer content was found to scale with the average layer number [42,46], a plot of PL/Raman ratio as function of layer number should reveal variations in PLQY in the different batches of LPE WS_2_. These are expected for the following reasons. First, the different length to thickness ratios, or more precisely, the characteristic monolayer length should be reflected in the PL/Raman ratio. This is because the contribution of nanosheet edges, which do not show the A-exciton PL [31], is larger in smaller nanosheets. Second, the density of defects on the basal plane of the exfoliated nanosheets should have an impact on the fluorescence intensity. While point defects in semiconductors can act as exciton traps resulting in redshifted, relatively intense PL [63,67,68,69], many defects in TMDs were reported to be detrimental for the emission [63,70,71]. In addition, we note that we did not observe additional photoluminescence from midgap states at energies below the A-exciton so that we believe a larger basal plane defect density should result in quenched PL in our types of samples.

The PL/Raman intensity ratio is shown in Figure 4E,F. The data follows an empirical power law. Within the 30% amplitude series (Figure 4E) the PL/Raman ratios of Ref 30% lie clearly below the PL/Raman ratios of the pretreated materials, especially for low <N>. The pretreated batches group together, but differences are present. A similar observation was made for the 60% amplitude series (Figure 4F), where the PL/Raman ratios of NaCl stir 60% exceed Ref 60%. Due to the efficient exfoliation with a 60% amplitude without pretreatment, this effect is less pronounced as in the 30% amplitude series, but clearly discernible. This suggests that the pretreatment improves the optical quality of the WS_2_ nanosheets in terms of photoluminescence quantum yield.

**Figure 5:**
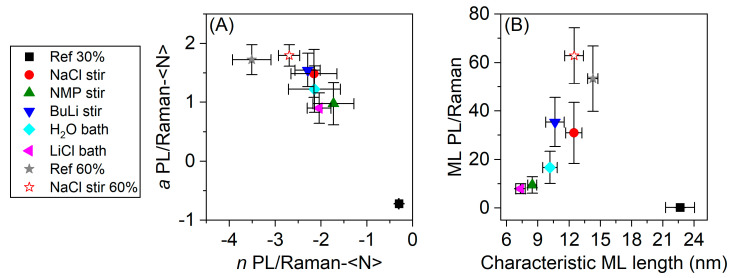
(**A**) Prefactors and exponents extracted from the fits shown in Figure 4. The highest prefactors were found for NaCl stir 60%, closely followed by Ref 60%. The pretreated materials of the 30% series group together. Ref 30% shows the lowest prefactor and smallest value of the exponent. (**B**) ML PL/Raman as a function of the characteristic ML length. The higher the ML length, the higher the ML PL/Raman ratio, with exception of Ref 30%, which shows a negligible ML PL/Raman ratio despite of the highest characteristic ML length, suggesting defective nanosheets.

To visualize this effect more clearly, we fit the data in Figure 4E,F to powerlaws to extract the exponents, *n*, and prefactors, 10*^a^*. The fit parameters are plotted versus each other in Figure 5A and reveal a correlation. As with the <L>–<N> fits above, the prefactor has a physical meaning, as it represents the PL/Raman ratio of a theoretical pure ML dispersion with <N> = 1 and is thus a measure for the relative PLQY. A low prefactor of the fit implies that the PL remains negligible for small <N>. Ref 30% is the only batch with a negative prefactor fit parameter. The prefactor fit parameter of the other batches are positive and group together. The highest prefactor is found in the batch NaCl stir 60%, closely followed by Ref 60%. In Figure 5B, the extrapolated PL/Raman ratio of a pure ML dispersion is plotted against the characteristic ML length of the dispersion which was extracted from the <L> versus <N> data. Within the pretreated WS_2_ starting materials of the 30% series, we find that the PL/Raman ratio indeed increases with the characteristic ML length as one would expect when considering that edges are sites of nonradiative exciton decay. The batches exfoliated at 60% amplitude have a slightly higher characteristic ML length and significantly higher ML PL/Raman ratio. NaCl stir 60% even outperforms Ref 60% with respect to the PL/Raman ratio, despite of the slightly lower ML length. Importantly, despite the highest characteristic ML length, Ref 30% shows only negligible PL which is in line with the hypothesis presented above that these nanosheets are defective on the basal plane. The pretreatment facilitated the exfoliation by lowering the interlayer binding energy resulting in exfoliation of less defective nanosheets in the order LiCl bath < NMP stir < H_2_O bath < NaCl stir < BuLi stir. Within this pretreatment series, the PL/Raman ratio seems to be limited by the characteristic ML length. The optical properties of Ref 60% are superior to pretreated materials of the 30% amplitude series. Due to the higher amplitude, defect-free areas of the bulk material were exfoliated without pretreatment. NaCl stir 60% shows a slightly improved PL/Raman ratio compared to Ref 60%, demonstrating that a small effect of the pretreatment remains under our standard exfoliation conditions.

## 5. Conclusions

In summary, we investigated the effect of different pretreatment conditions of the bulk powder prior to LPE using WS_2_ as model system. Particular emphasis was placed on the length/thickness aspect ratio and characteristic monolayer size, respectively since this should be a measure for the ratio of interlayer to intralayer binding strength according to a recent model of the exfoliation [46]. It was expected that intercalation as a result of the pretreatment would lower the interlayer binding strength thus resulting in an increase of the characteristic monolayer length. However, for low amplitude sonication, the opposite was observed, i.e., the characteristic monolayer length of the nanosheets in the reference samples was larger than when exfoliating pretreated powder. This can be explained by efficient exfoliation of only defective nanosheets which have an intrinsically lower interlayer binding energy. This hypothesis was strongly supported by photoluminescence measurements which revealed a scaling of the PLQY with characteristic monolayer size only when pretreated powders were used or exfoliation was performed with high amplitude. Overall, the study shows that pretreatment, for example stirring in aqueous NaCl, is capable of reducing the interlayer binding strength in layered crystals to some extent. While the effect is not very pronounced and no significant change in the length/thickness aspect ratio is achieved, the photoluminescence quantum yield can be improved. The study gives important insights in the fundamentals of the exfoliation and emphasizes that the role of defects in LPE, especially defects present in the starting powder, merits more attention.

## Data Availability

Correspondence and requests for data and materials should be addressed to C.B.
